# Residue-Level Energetic Analysis of Transient Misfolding in the Whirlin PDZ Tandem Reveals a Possible Connection to an AlphaFold-Predicted Alternative Topology

**DOI:** 10.3390/ijms27198509

**Published:** 2026-09-24

**Authors:** Valeria Pennacchietti, Mariana Di Felice, Julian Toso, Eduarda Santos Ventura, Laura Caldarelli, Angelo Toto, Francesca Malagrinò, Stefano Gianni

**Affiliations:** 1 Laboratory Affiliated to Istituto Pasteur Italia, Fondazione Cenci Bolognetti, Dipartimento di Scienze Biochimiche “A. Rossi Fanelli”, Sapienza Università di Roma, P.le Aldo Moro 5, 00185 Rome, Italy; 2 Dipartimento di Medicina Clinica, Sanità Pubblica, Scienze Della Vita e Dell’ambiente, Università dell’Aquila, Piazzale Salvatore Tommasi 1, Coppito, 67010 L’Aquila, Italy

**Keywords:** kinetics, misfolding, mutagenesis, tandem domains

## Abstract

Multidomain proteins may populate transient misfolded states that compete with productive folding, but the molecular determinants of these intermediates remain poorly understood. Using the tandem PDZ domains of the scaffolding protein whirlin, in which a misfolded intermediate accumulates only when both domains are denatured, we combined systematic mutagenesis with a thermodynamic framework analogous to double-mutant cycle analysis. Analysis of ~70 mutations identified residues displaying significant non-additive energetic effects, defining an interaction network concentrated within the connecting hairpins, PDZ2, and a few positions at the PDZ1 interface. Interestingly, AlphaFold predicted an alternative domain-swapped topology differing from the experimentally determined structure primarily within these same regions. Although the experimental data do not establish the structure of the misfolded intermediate, this correspondence suggests that the predicted alternative topology may provide a possible structural interpretation of the experimentally identified energetic determinants. Our findings provide a residue-level characterization of the energetic determinants of transient misfolding in a multidomain protein and suggest a possible connection between these determinants and alternative structural arrangements.

## 1. Introduction

Protein misfolding is traditionally discussed in the context of aggregation, where non-native conformations are stabilized through intermolecular interactions that lead to oligomers or amyloid assemblies [1,2,3]. While this framework has provided fundamental insight into protein homeostasis and disease, it has also focused attention on aggregated species, leaving comparatively little known about transient monomeric misfolded states that arise during folding. These states are particularly challenging to characterize because they are short-lived, structurally heterogeneous, and rarely accumulate to detectable levels under equilibrium conditions [4,5,6,7,8,9,10]. Yet, they may play an important role in shaping folding pathways by competing with productive routes to the native state.

Multidomain proteins provide a particularly attractive system in which to investigate monomeric misfolding. Because multiple domains fold within a single polypeptide chain, transient non-native interactions can occur during folding and potentially stabilize alternative conformations [4,11,12,13]. The tandem PDZ domains of the scaffolding protein whirlin represent a well-defined model to explore this phenomenon. Previous studies have shown that, while the first PDZ domain folds independently when the second domain is natively structured, simultaneous denaturation of both domains gives rise to a transient misfolded intermediate that slows folding and competes with the native state [7,8]. Despite its clear kinetic signature, the structural and energetic determinants underlying this monomeric misfolded state remain unknown.

Here, we address this problem by combining systematic protein engineering with a thermodynamic framework formally analogous to double mutant cycle analysis [14,15,16,17]. By comparing the effects of site-directed mutations under conditions where the second PDZ domain is either folded or unfolded, we identify residues that are energetically coupled to the formation of the misfolded intermediate. Mapping these residues onto the structure reveals a defined interaction network concentrated in specific regions of the tandem. We then compare this experimentally derived energetic map with structural models generated by AlphaFold, uncovering an unexpected correspondence between the determinants of misfolding and an alternative topology predicted by the algorithm.

## 2. Results and Discussion

To characterize the folding behavior of the PDZ tandem, we first analyzed the refolding kinetics under conditions that selectively perturb the two domains. When refolding was initiated from 2.2 M GdnHCl, where only PDZ1 is unfolded and PDZ2 remains in its native conformation, the observed chevron plot displayed a canonical profile, consistent with a three-state-like folding mechanism (Figure 1). In contrast, when the protein was unfolded at high denaturant concentration (5.5 M GdnHCl), such that both PDZ domains are denatured, the refolding branch exhibited a much more pronounced roll-over with the folding rate constant increasing with increasing denaturant concentration. This finding, as discussed earlier on this and other protein systems [5,7,9,10], is indicative of the accumulation of a misfolded intermediate that competes with productive folding. Furthermore, this behavior confirms that the formation of the misfolded state requires the simultaneous unfolding of both domains and is not accessible when PDZ2 is maintained in its native conformation.

To dissect the energetic determinants underlying the formation of the misfolded intermediate, we performed a systematic mutational analysis spanning the whole tandem. For each variant, refolding kinetics were measured under both conditions described above, enabling direct comparison between folding in the absence and presence of the misfolded state. This experimental design can be formally interpreted as a double perturbation scheme [14,15,16,17], where the first perturbation is the site-directed mutation and the second perturbation is the denaturation of PDZ2. Under this framework, residues whose mutational effects differ between the two conditions display a non-additive response, corresponding to a detectable ΔΔΔ*G* effect. Such residues are therefore energetically coupled to the formation of the misfolded intermediate.

Analysis of approximately 70 site-directed variants revealed that a subset of residues exhibits a significant ΔΔΔ*G* signal, indicating their specific involvement in stabilizing or modulating the misfolded state (Figures S1 and S2; Table 1). Following standard practice in double-mutant cycle analysis [14,15,16,17], only |ΔΔΔG| values exceeding 0.4 kcal mol^−1^ were considered indicative of meaningful energetic coupling. Residues displaying such coupling were then mapped onto the experimentally restrained structural model of the PDZ1–PDZ2 tandem reported by Delhommel et al. [18] to examine their spatial distribution. This structural model was obtained using an integrative approach in which the structures of the individual PDZ domains were combined with experimental restraints defining their supramodular arrangement, including RDCs, PREs and NMR-derived dihedral-angle restraints, and was subsequently validated by SAXS, HDX-MS and mutational/NMR analyses. The coordinates used here were kindly provided directly by the authors of the original study, as the tandem model was not deposited as a separate PDB entry. As in conventional double-mutant-cycle analysis, the magnitude and sign of ΔΔΔG provide complementary information. Values close to zero indicate that the energetic effect of a mutation is essentially unchanged between the two folding regimes, whereas significant positive or negative values indicate that the energetic consequence of the mutation depends on whether the misfolded pathway is accessible. The sign reflects the direction of this energetic effect, while |ΔΔΔG| reports the magnitude of the non-additivity. Accordingly, identification of residues energetically coupled to misfolding was based primarily on |ΔΔΔG| rather than on its sign.

The resulting distribution was highly non-random. Whereas only 4 of the 24 positions analyzed in PDZ1 (17%) displayed significant coupling, the corresponding fraction increased to 10 of 18 positions (56%) within the two hairpins and 13 of 27 positions (48%) in PDZ2 (Figure 2). Importantly, this regional enrichment does not imply that every residue within these structural elements should display detectable energetic coupling, since the analysis identifies individual side chains whose energetic contributions differ between the productive and misfolded folding regimes. Notably, the few coupled positions detected in PDZ1 are located near the interface connecting the domain to the adjacent structural elements (see Figure 2A). This pattern indicates that the energetic signature of the misfolded state is concentrated within a defined interaction network involving PDZ2 and the two connecting hairpins rather than being distributed throughout the tandem. Furthermore, the non-uniform distribution suggests that the misfolded intermediate is stabilized by specific interactions and structural rearrangements rather than by nonspecific collapse. Because the energetic analysis reports only on contributions that differ from those observed during productive folding, the enrichment of coupled residues in the hairpins and PDZ2 identifies the regions that contribute most strongly to the alternative pathway leading to misfolding. Importantly, this spatial enrichment emerged from the experimental energetic analysis independently of any structural model and was only subsequently compared with the AlphaFold prediction.

To investigate whether this experimentally defined interaction network could be associated with a specific structural rearrangement, we analyzed AlphaFold [19] predictions generated from the complete sequence of the PDZ tandem construct. Five models were generated, and all five reproduced the same alternative topology, although some variability was observed in the precise relative arrangement of the structural elements. The models displayed very similar global confidence metrics, with pTM values of 0.52–0.53 and ranking scores of 0.60–0.62 (Table S1). Interestingly, comparison with the experimentally restrained structural model of Delhommel et al. [18] revealed that the predicted alternative topology differs primarily in the organization of the hairpins and PDZ2, while PDZ1 remains largely conserved. The AlphaFold models display an alternative domain-swapped topology in which the hairpin-mediated interdomain connectivity is reorganized (Figure 2B). Per-residue pLDDT values and PAE analyses are shown in Figure S3 and Figure S4, respectively [19].

**Figure 2:**
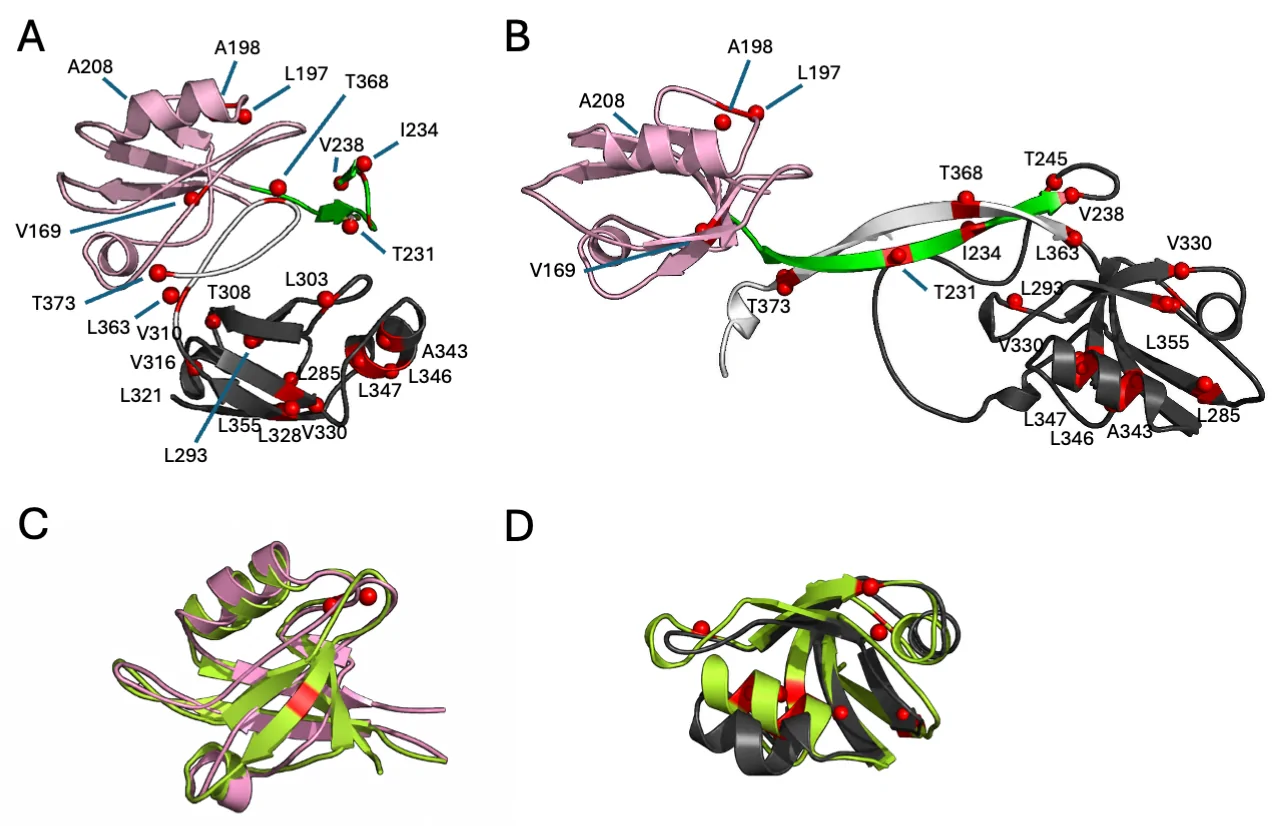
Structural comparison between the experimentally restrained structural model of the Whirlin PDZ1–PDZ2 tandem reported by Delhommel et al. (panel (**A**)) and the AlphaFold 3 model of the same supramodular region (panel (**B**)). In both structures, PDZ1 is depicted in pink, hairpin1 in green, PDZ2 in black and hairpin2 in white. Residues displaying |ΔΔΔG| ≥ 0.4 kcal mol^−1^ are highlighted with a red sphere in both structures. Panels (**C**,**D**) highlight the structural superposition of PDZ1 and PDZ2, respectively. In both panels the Alphafold model is depicted in light green. It is evident that, whilst PDZ1, displays a high degree of structural conservation with some structural rearrangements at the level of the front b-sheet (i.e., at the interface with hairpin1), PDZ2 highlights more noticeable structural differences.

Remarkably, the structural elements highlighted by the ΔΔΔG analysis correspond to those most strongly reorganized in the AlphaFold model. Structural superposition shows that PDZ1 is largely conserved between the two structures, whereas the largest deviations are confined to PDZ2 and the connecting hairpins (Figure 2C,D). Thus, the experimentally derived energetic map and the independently generated structural prediction highlight the same regions of the tandem, although the AlphaFold topology should be regarded as a possible structural interpretation of the energetic pattern rather than as a direct structural determination of the kinetic intermediate. The present study addresses a longstanding challenge in protein folding: the characterization of transient monomeric misfolded states [1,2,3,20,21,22]. Unlike aggregated species, which can often be isolated and studied directly, monomeric misfolded intermediates are typically short-lived and only transiently populated, making their structural determinants difficult to define experimentally. By exploiting a multidomain system in which the misfolded state can be selectively populated, we identified a discrete set of residues that contribute specifically to the alternative folding pathway, providing a residue-level description of a transient misfolded state.

A striking aspect of the analysis is its connection to domain swapping. Domain-swapped architectures have frequently been invoked to explain alternative protein conformations and misfolded states, yet direct experimental support has remained limited [5,6,7,23,24,25]. In the case of the whirlin PDZ tandem, the energetic determinants of misfolding localize within the same structural elements that are reorganized in an alternative domain-swapped topology predicted by AlphaFold. Although the present data do not establish the structure of the misfolded intermediate directly, they are consistent with the possibility that transient domain swapping underlies the observed kinetic trap. The fact that both PDZ domains remain largely folded in the AlphaFold model does not exclude such a kinetic mechanism, since trapping does not require global unfolding but rather the formation of alternative interactions that must be disrupted before productive folding can proceed. Experimentally, these interactions can only become accessible when PDZ2 is initially unfolded, consistent with the absence of the pronounced misfolding phase when refolding is initiated from conditions in which PDZ2 remains native. Additional experimental evidence indicates that the hairpin extensions possess an intrinsic propensity to form β-structure. Analysis of the NMR chemical shifts reported by Delhommel et al. showed that, in addition to the canonical secondary structure of the two PDZ domains, the 18 residues following the C termini of the two domains display backbone torsion angles characteristic of β-strand conformations. These extensions were also shown experimentally to contribute to formation of the native PDZ1–PDZ2 supramodule [18]. Although this observation does not establish the topology of the transient misfolded intermediate, it supports the structural competence of these regions to participate in alternative β-mediated interactions.

More generally, our findings suggest that transient monomeric misfolding can emerge from specific sequence-encoded interactions embedded within otherwise functional multidomain proteins. The correspondence between the experimentally derived energetic map and the alternative AlphaFold topology further raises an intriguing possibility: in some cases, discrepancies between predicted and experimentally determined structures may not simply reflect prediction errors but instead reveal alternative conformations encoded within the protein energy landscape. Thus, beyond providing insight into the folding mechanism of the whirlin PDZ tandem, the present work highlights how the combination of experiment and structure prediction may help uncover elusive conformational states that have remained largely inaccessible to direct structural characterization.

## 3. Materials and Methods

### 3.1. Protein Expression and Purification

The construct corresponded to the previously characterized mouse PDZ1–PDZ2 supramodule [7,8]. DNA encoding P1–P2 pWT and variants was transformed into Escherichia coli BL21 (DE3) cells for protein expression. The protein expression was induced with isopropyl β-D-1-thio-galactopyranoside to a final concentration of 100 μg/mL at an OD 600 of 0.8, and the cells were incubated for 3 h at 30 °C, and overnight at 20 °C. The cells were then cultured. Each construct and mutant was purified from the soluble fraction. Cell pellets were resuspended in lysis buffer containing 50 mM Tris-HCl pH 7.5, 300 mM NaCl, and protease inhibitors, and disrupted by sonication. The soluble fraction was clarified by centrifugation and purified by affinity chromatography followed by size-exclusion chromatography, as described previously [7,8]. The purity of the protein sample was confirmed by sodium dodecyl sulfate polyacrylamide gel electrophoresis.

Mutations were designed to provide broad coverage of the entire PDZ1–PDZ2 tandem, including PDZ1, the hairpin/linker regions and PDZ2, rather than being selected on the basis of the AlphaFold prediction. Conservative substitutions were used wherever possible to perturb individual side-chain interactions while minimizing disruption of the overall fold, using the standard rule of double mutant cycles [15,16]. The mutational dataset was therefore generated independently of the subsequent AlphaFold structural comparison.

### 3.2. Kinetic Folding Experiments

Stopped-flow kinetic experiments were performed using an SX-18MV stopped-flow apparatus (Applied Photophysics, Leatherhead, UK) by monitoring intrinsic tryptophan fluorescence. Refolding and unfolding reactions were initiated by rapid mixing of protein samples with buffers containing different concentrations of guanidinium hydrochloride (GdnHCl). Refolding experiments were performed either from mildly denaturing conditions, in which only PDZ1 is unfolded (2.2 M GdnHCl), or from strongly denaturing conditions, where both PDZ domains are unfolded (>5 M GdnHCl). Unless otherwise stated, experiments were carried out at 25 °C in 50 mM Tris-HCl pH 7.5, and 0.5 M Na_2_SO_4_ was added as a stabilizing agent to enhance accumulation of the misfolded intermediate. Fluorescence emission was collected using a cut-off filter above 320 nm following excitation at 280 nm. Each experiment represents the average of at least six individual acquisitions. The averaged kinetic traces were satisfactorily fitted using single-exponential functions, and the observed rate constants were analyzed as a function of denaturant concentration to generate chevron plots. Kinetic parameters were obtained by global fitting of the chevron plots using shared m-values, and the errors reported in Table 1 correspond to the standard errors obtained from the global fits.

### 3.3. Data Analysis

The chevron plots were fitted using the following equations:

$$log{\left(k_{obs}\right)}_{1}=log\left(\frac{\left({\;k}_{Fmis}\;\cdot e^{{{-m}_{F}}^{\cdot \left[GndHCl\right]}}\right)}{\left(1+K_{mis}\cdot e^{{-m}_{mis\;}\cdot \left[GndHCl\right]}\right)}+k_{U\;}\cdot e^{m_{U}\cdot \left[GndHCl\right]}\right)$$

$$log{\left(k_{obs}\right)}_{2}=log\left(\frac{\left({\;k}_{Fmis}\;\cdot e^{{{-m}_{F}}^{\cdot \left[GndHCl\right]}}\right)}{\left(1+K_{mis2.2M}\cdot e^{{-m}_{mis2.2M\;}\cdot \left[GndHCl\right]}\right)}+k_{U\;}\cdot e^{m_{U}\cdot \left[GndHCl\right]}\right)$$

The thermodynamic parameters were calculated as follows:

∆∆G_D-I_ = (RT · ln(*K*_D-Iwt_)) − (RT · ln(*K*_D-Imut_))

∆∆G_D-MIS_ = (RT · ln(*K*_D-MISwt_)) − (RT · ln(*K*_D-MISmut_))

∆∆∆G= ∆∆G_D-I_ − ∆∆G_D-MIS_

### 3.4. AlphaFold Structure Prediction

Structure prediction was performed using AlphaFold 3 on the complete sequence of the PDZ1–PDZ2 construct. Five models were generated using 10 recycles. All five models reproduced the alternative topology discussed in the text, with some variability in the precise relative arrangement of the structural elements. Global confidence was similar across the five predictions, with pTM values ranging from 0.52 to 0.53 and ranking scores from 0.60 to 0.62. Prediction confidence was further assessed using per-residue pLDDT values and predicted aligned error (PAE), reported in Figure S3 and Figure S4, respectively; global confidence metrics for the five models are summarized in Table S1. Predictions were performed only for the WT PDZ1–PDZ2 construct; individual mutant variants were not modeled, as the mutational analysis was used experimentally to define the energetic determinants of misfolding rather than to generate mutant-specific structural predictions.

## Figures and Tables

**Figure 1:**
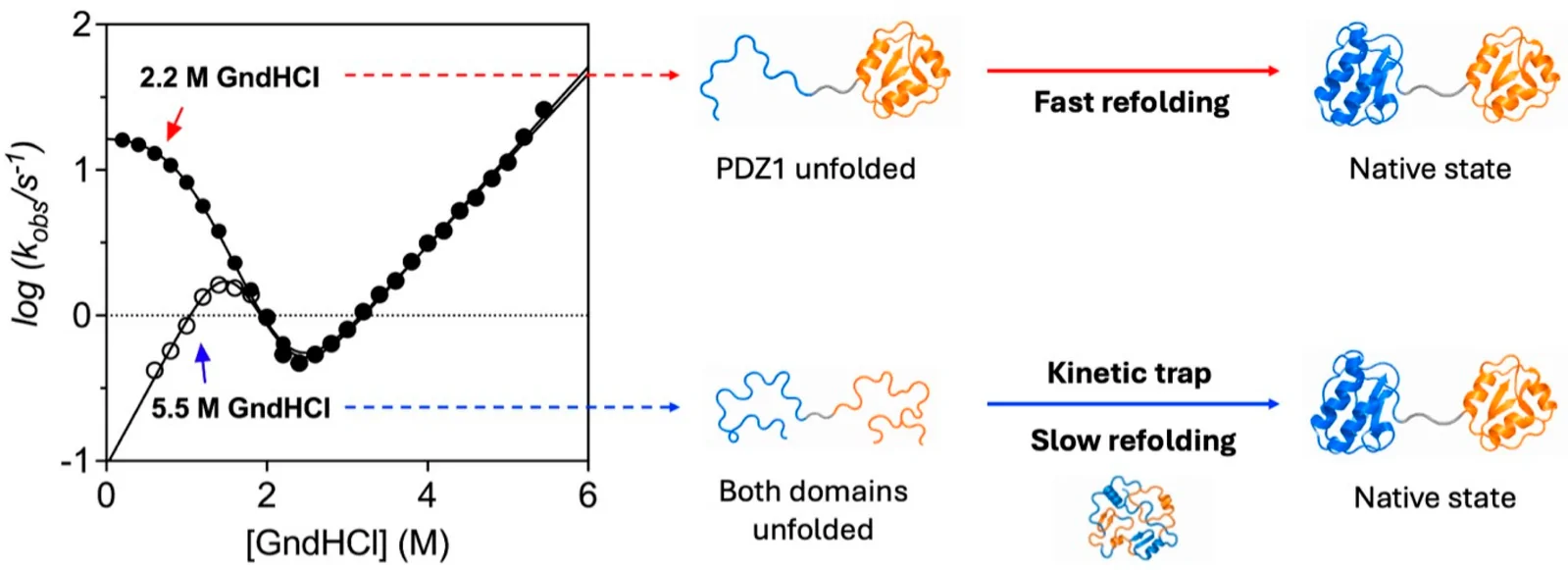
Schematic representation of the two refolding pathways observed for the Whirlin PDZ tandem under different denaturing conditions. When refolding is initiated from 2.2 M GdnHCl, only PDZ1 is unfolded while PDZ2 remains natively folded, resulting in a fast two-state-like refolding pathway directly leading to the native state. In contrast, when refolding starts from a high denaturant concentration (5.5 M GdnHCl), both PDZ domains are unfolded and the refolding branch displays markedly slower kinetics due to the accumulation of a misfolded intermediate. In this condition, transient non-native interactions between the two unfolded domains generate a kinetic trap that competes with productive folding before reaching the native state. The corresponding chevron plots highlight the distinct refolding behaviors observed under the two experimental conditions.

**Table 1:** Thermodynamic parameters of PDZ1-PDZ2 variants.

Variant	Position	K_D-I_	K_D-mis_	ΔΔG_D-I_ [kcal mol ^−1^]	ΔΔG_D-mis_ [kcal mol^−1^]	ΔΔΔG [kcal mol^−1^]
WT	-	22.0 ± 1.0	4340 ± 220	-	-	-
L141A	PDZ1	20.0 ± 1.2	7400 ± 630	0.07 ± 0.05	−0.60 ± 0.05	−0.37 ± 0.05
A147G	PDZ1	31.0 ± 4.0	6620 ± 480	−0.20 ± 0.02	−0.25 ± 0.02	−0.05 ± 0.03
L153A	PDZ1	13.0 ± 1.2	3080 ± 360	0.30 ± 0.03	0.20 ± 0.06	−0.11 ± 0.07
I157V	PDZ1	20.0 ± 2.2	3890 ± 300	0.07 ± 0.03	0.07 ± 0.07	−0.0 ± 0.08
V165A	PDZ1	26.0 ± 2.0	6920 ± 500	−0.10 ± 0.03	−0.27 ± 0.07	−0.18 ± 0.07
I167V	PDZ1	29.0 ± 3.0	5390 ± 480	−0.20 ± 0.03	−0.13 ± 0.09	0.02 ± 0.10
V169A *	PDZ1	0.80 ± 0.03	60 ± 4	2.00 ± 0.03	2.50 ± 0.06	0.50 ± 0.06
L171A	PDZ1	39.0 ± 4.0	12,550 ± 1090	−0.30 ± 0.03	−0.60 ± 0.05	−0.30 ± 0.06
L177A	PDZ1	6.4 ± 1.3	2020 ± 220	0.70 ± 0.06	0.45 ± 0.09	−0.29 ± 0.10
A178G	PDZ1	1.00 ± 0.04	150 ± 10	1.90 ± 0.03	2.00 ± 0.10	0.10 ± 0.10
V185A	PDZ1	11.0 ± 1.1	3870 ± 320	0.40 ± 0.03	0.07 ± 0.01	−0.35 ± 0.03
I189V	PDZ1	24.0 ± 2.0	6140 ± 440	−0.04 ± 0.05	−0.20 ± 0.02	−0.16 ± 0.06
L190A	PDZ1	12.0 ± 1.6	3730 ± 200	0.40 ± 0.03	0.09 ± 0.03	−0.28 ± 0.04
V192A	PDZ1	22.5 ± 1.4	4540 ± 220	−0.10 ± 0.05	−0.03 ± 0.10	−0.02 ± 0.10
L197A *	PDZ1	4.0 ± 0.2	250 ± 10	1.0 ± 0.06	1.70 ± 0.05	0.67 ± 0.07
A198G *	PDZ1	23.0 ± 1.8	14,370 ± 980	−0.02 ± 0.05	−0.70 ± 0.06	−0.70 ± 0.06
V200A	PDZ1	12.0 ± 1.2	3380 ± 480	0.40 ± 0.03	0.15 ± 0.06	−0.22 ± 0.06
A203G	PDZ1	12.0 ± 2.0	2440 ± 280	0.40 ± 0.10	0.34 ± 0.03	−0.03 ± 0.12
A205G	PDZ1	7.7 ± 1.0	1810 ± 160	0.60 ± 0.05	0.50 ± 0.04	−0.10 ± 0.01
V206A	PDZ1	11.0 ± 1.8	2940 ± 500	0.40 ± 0.03	0.23 ± 0.02	−0.19 ± 0.03
A208G *	PDZ1	13.0 ± 0.8	780 ± 80	0.30 ± 0.03	1.00 ± 0.08	0.70 ± 0.09
L215A	PDZ1	20.0 ± 3.0	2120 ± 330	0.07 ± 0.04	0.40 ± 0.03	0.35± 0.03
V216A	PDZ1	10.0 ± 2.0	1200 ± 200	0.50 ± 0.04	0.77 ± 0.06	0.30 ± 0.02
V230A	PDZ1	22.0 ± 1.3	4980 ± 10	0.01 ± 0.10	−0.08 ± 0.03	−0.09 ± 0.01
T231S *	Hairpin1	23.0 ± 2.2	12,650 ± 1000	−0.02 ± 0.10	−0.60 ± 0.05	−0.60 ± 0.05
I234V *	Hairpin1	25.0 ± 1.7	10,160 ± 700	−0.07 ± 0.01	−0.50 ± 0.04	−0.40 ± 0.03
T236S	Hairpin1	33.0 ± 3.0	5430 ± 370	−0.20 ± 0.02	−0.10 ± 0.03	0.10 ± 0.03
V238A *	Hairpin1	41.0 ± 5.0	19,100 ± 2000	−0.40 ± 0.03	−0.90 ± 0.07	−0.50 ± 0.04
T245S *	Hairpin1	17.0 ± 1.6	6840 ± 460	0.20 ± 0.03	−0.27 ± 0.02	−0.40 ± 0.03
L251A	Hairpin1	25.0 ± 3.0	7190 ± 630	−0.07 ± 0.03	−0.30 ± 0.02	−0.22 ± 0.02
T258S	Hairpin1	27.0 ± 3.0	8130 ± 710	−0.10 ± 0.03	−0.37 ± 0.03	−0.26 ± 0.02
L259A	Hairpin1	40.0 ± 6.0	8490 ± 530	−0.30 ± 0.03	−0.40 ± 0.03	−0.05 ± 0.03
L270A	PDZ2	29.0 ± 3.0	7770 ± 610	−0.20 ± 0.03	−0.34 ± 0.03	−0.20 ± 0.02
L272A	PDZ2	40.0 ± 4.0	5710 ± 540	−0.30 ± 0.03	−0.20 ± 0.05	0.20 ± 0.05
L273A	PDZ2	37.0 ± 3.0	4990 ± 320	−0.30 ± 0.02	−0.08 ± 0.05	0.21 ± 0.06
V281A	PDZ2	25.0 ± 2.4	7120 ± 550	−0.07 ± 0.03	−0.30 ± 0.02	−0.20 ± 0.02
L283A	PDZ2	21.0 ± 3.0	4590 ± 500	0.04 ± 0.10	−0.03 ± 0.10	−0.07 ± 0.01
V284A	PDZ2	21.0 ± 3.0	6110 ± 650	0.04 ± 0.10	−0.20 ± 0.02	−0.20 ± 0.02
L285A *	PDZ2	19.0 ± 2.2	8030 ± 650	0.10 ± 0.03	−0.40 ± 0.03	−0.45 ± 0.04
L291A	PDZ2	26.0 ± 3.0	4840 ± 420	−0.09 ± 0.03	−0.07 ± 0.06	0.02 ± 0.10
L293A *	PDZ2	22.0 ± 4.0	10,460 ± 1080	0.01 ± 0.10	−0.50 ± 0.04	−0.50 ± 0.04
A299G	PDZ2	30.0 ± 3.0	6660 ± 590	−0.20 ± 0.03	−0.30 ± 0.02	−0.08 ± 0.03
L303A *	PDZ2	28.0 ± 1.9	2640 ± 150	−0.10 ± 0.04	0.30 ± 0.05	0.40 ± 0.03
I305V	PDZ2	29.0 ± 3.0	7120 ± 480	−0.20 ± 0.03	−0.30 ± 0.02	−0.10 ± 0.01
I307V	PDZ2	25.0 ± 3.0	5240 ± 520	−0.07 ± 0.04	−0.10 ± 0.05	−0.05 ± 0.06
T308S *	PDZ2	23.0 ± 1.3	11,860 ± 1080	−0.02 ± 0.10	−0.60 ± 0.05	−0.60 ± 0.05
V310A *	PDZ2	25.0 ± 1.6	16,080 ± 1680	−0.07 ± 0.03	−0.80 ± 0.06	−0.70 ± 0.06
A316G *	PDZ2	18.0 ± 1.0	6870 ± 560	0.10 ± 0.04	−0.30 ± 0.05	−0.40 ± 0.03
L321A *	PDZ2	23.0 ± 0.8	9710 ± 920	−0.02 ± 0.10	−0.47 ± 0.04	−0.45 ± 0.04
I327V *	PDZ2	26.0 ± 3.0	9810 ± 740	−0.09 ± 0.03	−0.50 ± 0.04	−0.40 ± 0.03
L328A *	PDZ2	26.0 ± 1.8	13,280 ± 1480	−0.09 ± 0.01	−0.66 ± 0.05	−0.57 ± 0.05
V330A *	PDZ2	25.0 ± 1.6	14,070 ± 1130	−0.07 ± 0.03	−0.70 ± 0.06	−0.60 ± 0.05
F335A	PDZ2	26.0 ± 1.0	8710 ± 960	−0.09 ± 0.03	−0.40 ± 0.03	−0.31 ± 0.03
L336A	PDZ2	26.0 ± 1.1	2960 ± 240	−0.09 ± 0.03	0.20 ± 0.05	0.30 ± 0.03
I338V	PDZ2	30.0 ± 4.0	8020 ± 730	−0.20 ± 0.02	−0.40 ± 0.03	−0.20 ± 0.02
L339A	PDZ2	32.0 ± 4.0	4510 ± 420	−0.20 ± 0.02	−0.02 ± 0.06	0.20 ± 0.06
A343G *	PDZ2	26.0 ± 3.0	10,160 ± 890	−0.09 ± 0.01	−0.50 ± 0.04	−0.40 ± 0.05
L346A *	PDZ2	25.0 ± 1.2	12,230 ± 900	−0.07 ± 0.03	−0.60 ± 0.05	−0.60 ± 0.04
L347A *	PDZ2	20.0 ± 1.1	15,420 ± 1340	0.07 ± 0.04	−0.75 ± 0.06	−0.80 ± 0.07
L353A	Hairpin2	23.0 ± 1.8	7120 ± 530	−0.02 ± 0.03	−0.30 ± 0.02	−0.30 ± 0.04
I354V	Hairpin2	22.0 ± 2.4	6690 ± 590	0.01 ± 0.04	−0.25 ± 0.02	−0.25 ± 0.04
L355A *	Hairpin2	24.0 ± 3.0	10,830 ± 980	−0.04 ± 0.03	−0.54 ± 0.04	−0.50 ± 0.04
L363A *	Hairpin2	22.0 ± 1.8	10,480 ± 1020	0.01 ± 0.02	−0.52 ± 0.04	−0.50 ± 0.04
T368S *	Hairpin2	26.0 ± 1.6	12,670 ± 1260	−0.09 ± 0.02	−0.63 ± 0.05	−0.50 ± 0.04
V369A	Hairpin2	26.0 ± 0.8	8410 ± 790	−0.09 ± 0.04	−0.40 ± 0.03	−0.30 ± 0.02
V370A *	Hairpin2	26.0 ± 1.2	9420 ± 860	−0.09 ± 0.02	−0.46 ± 0.04	−0.40 ± 0.04
T373S *	Hairpin2	25.0 ± 1.6	9440 ± 660	−0.06 ± 0.02	−0.46 ± 0.04	−0.40 ± 0.04
I376V *	Hairpin2	24.5 ± 1.2	9650 ± 760	−0.05 ± 0.03	−0.47 ± 0.04	−0.40 ± 0.03
I381V	Hairpin2	22.0 ± 1.0	6990 ± 670	0.01 ± 0.04	−0.28 ± 0.05	−0.30 ± 0.06

*K*_D-I_ and *K*_D-MIS_ denote the equilibrium constants associated with the equilibria between the denatured state and the intermediate and the denatured state and the misfolded state, respectively. Each kinetic trace was obtained by averaging at least six individual acquisitions. Kinetic parameters were obtained by globally fitting the data in Figures S1 and S2 with shared m-values; reported errors are standard errors from the global fits. A complete description of such analysis is reported in [8]. Mutants displaying a significant energetic coupling are marked with a *.

## Data Availability

The original contributions presented in this study are included in the article/Supplementary Material. Further inquiries can be directed to the corresponding authors.

## Supplementary Materials

The following supporting information can be downloaded at https://www.mdpi.com/article/10.3390/ijms27198509/s1.
