# Supplementary material for: Residue-Level Energetic Analysis of Transient Misfolding in the Whirlin PDZ Tandem Reveals a Possible Connection to an AlphaFold-Predicted Alternative Topology
Source: Int J Mol Sci. 2026 Sep 24;27(19):8509. doi: 10.3390/ijms27198509 (PMC13664925; doi:10.3390/ijms27198509)
Supplement: Supplementary file 1 [file ijms-27-08509-s001.zip › ijms-4544812-supplementary.pdf]

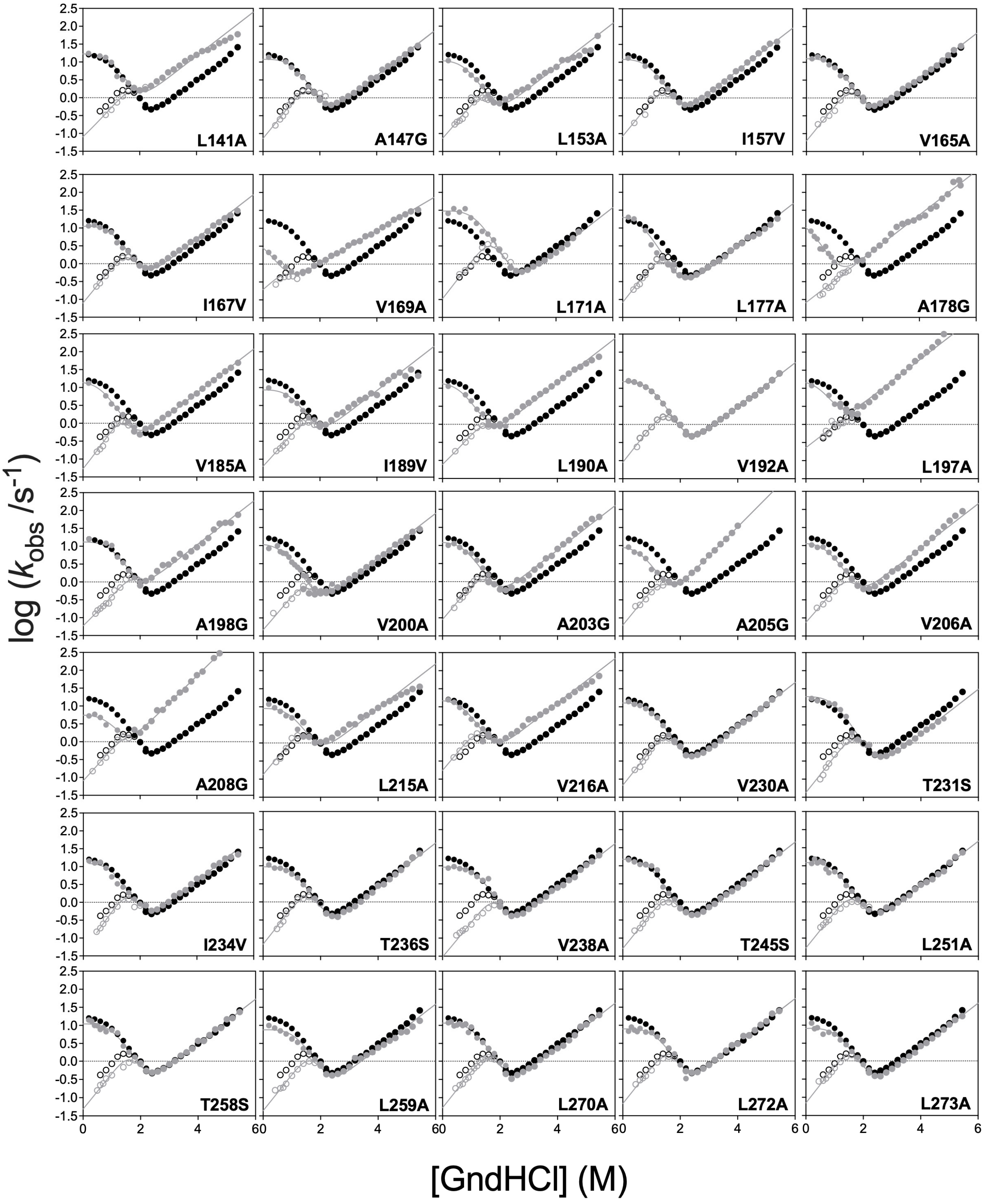

Figure S1. Comparison of kinetic chevron plots obtained for the wild-type PDZ1–PDZ2 tandem and for constructs carrying mutations in the PDZ1 domain. Both refolding branches were satisfactorily fitted across the investigated denaturant range, allowing quantitative analysis of the observed kinetics. Global fitting analysis was performed using shared *m*-values between wild-type and mutant constructs. The lines represent the best fit to the model described in the Materials and Methods section (open circles, Equation 1; filled circles, Equation 2).

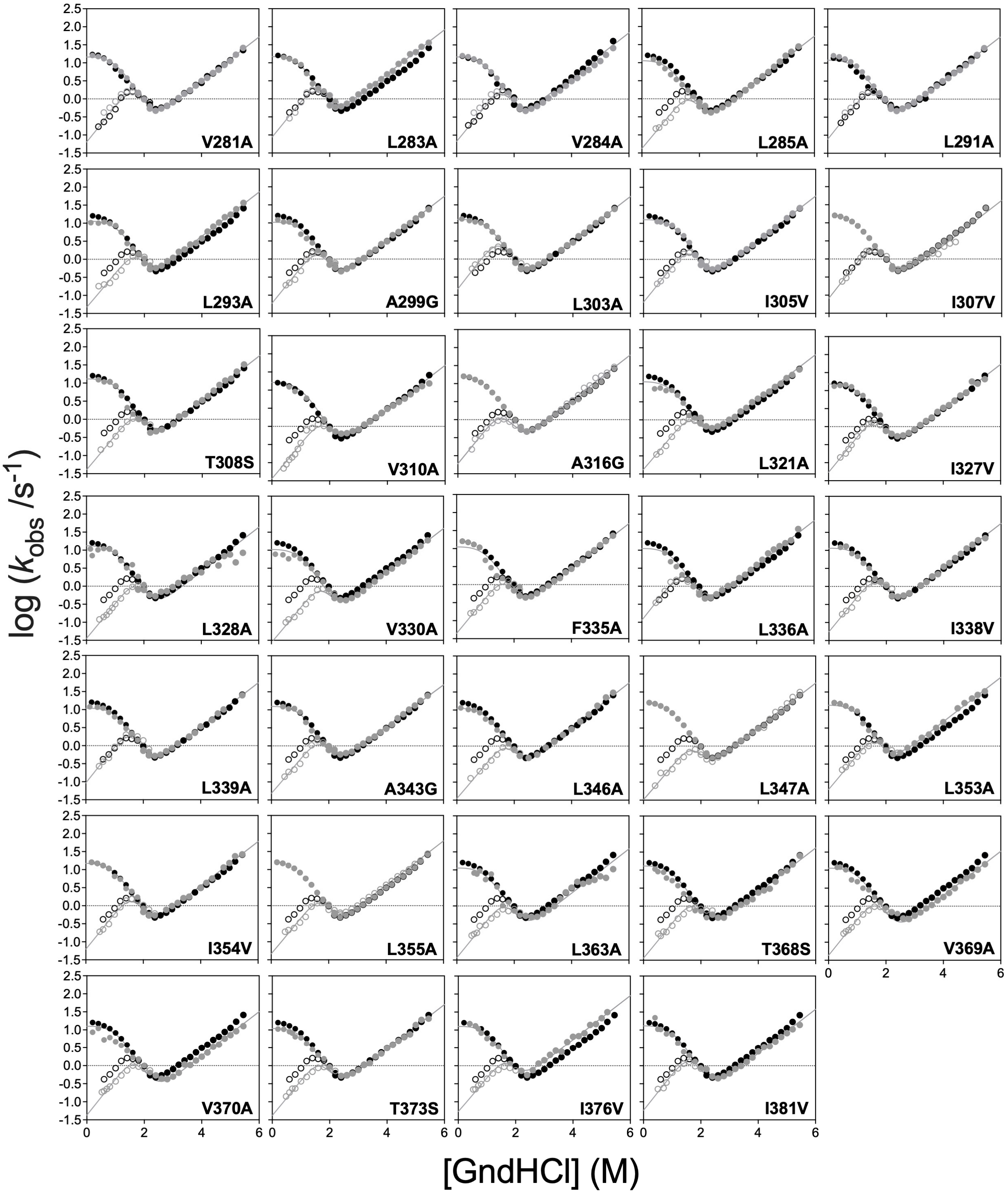

Figure S2. Comparison of kinetic chevron plots obtained for the wild-type PDZ1–PDZ2 tandem and for constructs carrying mutations in the PDZ2 domain. Both refolding branches were satisfactorily fitted across the investigated denaturant range, allowing quantitative analysis of the observed kinetics. Global fitting analysis was performed using shared *m*-values between wild-type and mutant constructs. The lines represent the best fit to the model described in the Materials and Methods section (open circles, Equation 1; filled circles, Equation 2).

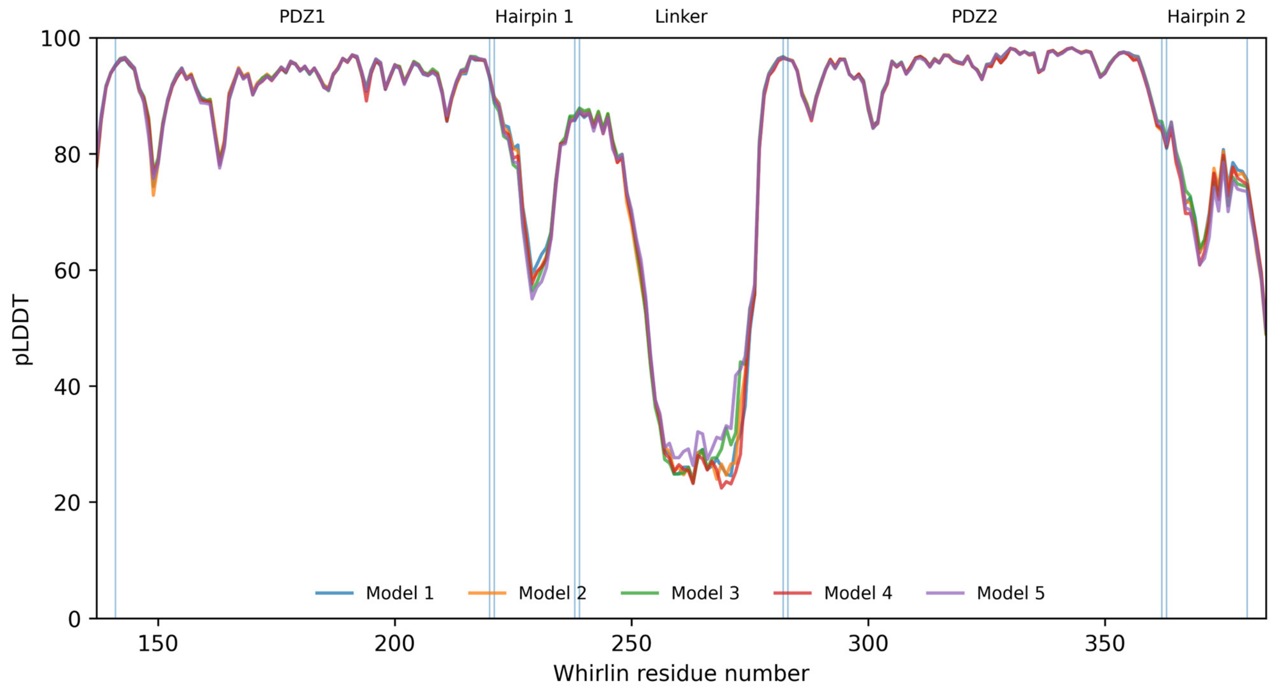

Figure S3. Per-residue confidence of the AlphaFold 3 predictions of the Whirlin PDZ1–PDZ2 tandem. Per-residue pLDDT values are shown for each of the five independently generated AlphaFold 3 models. All models display high local confidence within the canonical PDZ1 and PDZ2 domains, whereas lower confidence is observed in the connecting linker and, to a lesser extent, in the hairpin extensions. The approximate boundaries of PDZ1, hairpin 1, the interdomain linker, PDZ2 and hairpin 2 are indicated to facilitate comparison with the regions discussed in the main text. Importantly, pLDDT reports local structural confidence and should not be interpreted as a measure of confidence in the relative orientation of the two domains. The full protein sequence used in this study is
MGEVRLVSLRRAKAHEGLGFSIRGGSEHGVGIWVSLVEPGSLAEKEGLRVGDQILRVNDKSLARVTHAEAVKALKGSKKL
VLSVYSAGRIPGGYVTNHIYTWVDPQGRSTSPPSSLPQPHGSTLRQREDDRRSTLHLLQSGDEKKVNLVLGDGRSLGLTI
RGGAEYGLGIYITGVDPGSEAESSGLKVGDQILEVNGRSFLNILHDEAVKLLKSSRHLILTVKDVGRLPHARTTVDQTKWIA
SSRIGES

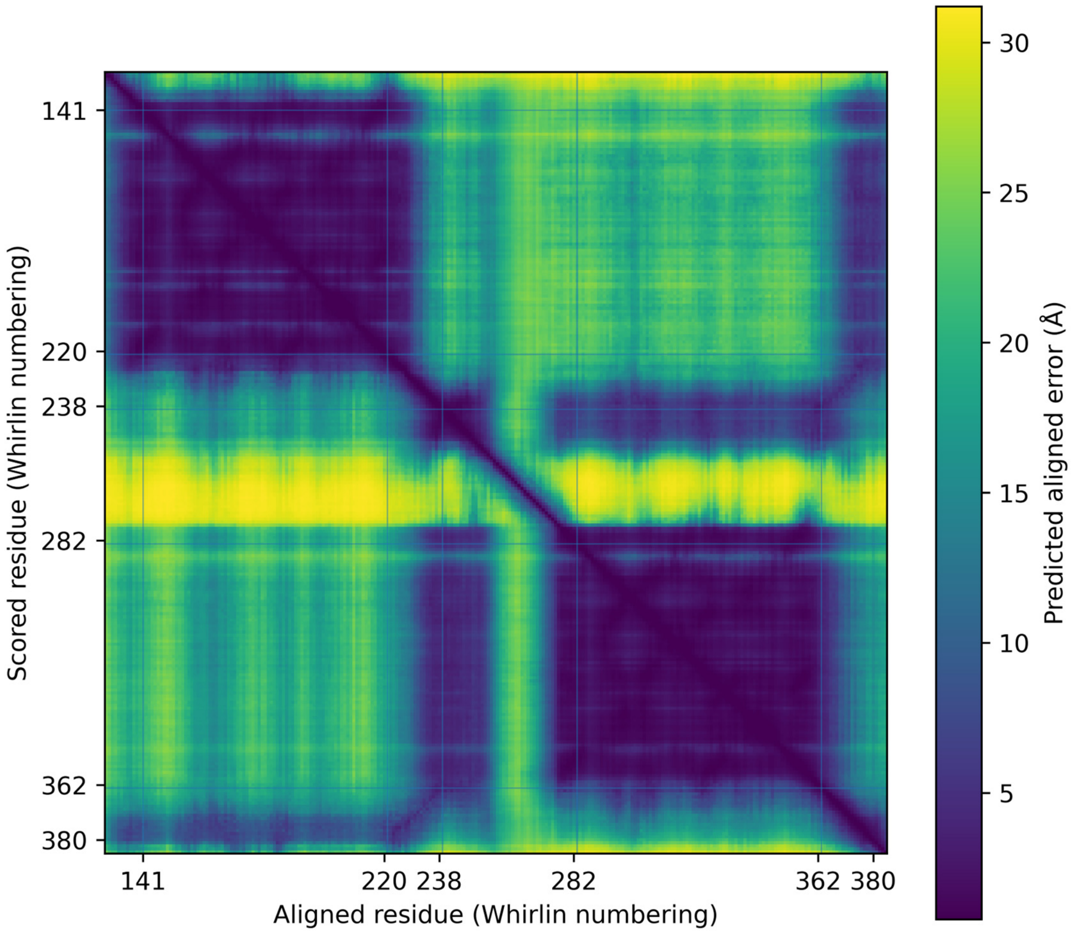

Figure S4. Predicted aligned error for the top-ranked AlphaFold 3 model of the Whirlin PDZ1–PDZ2 tandem. The PAE matrix is shown using Whirlin residue numbering. Low PAE values within PDZ1 and PDZ2 indicate high confidence in the internal geometry of the individual domains, whereas higher PAE values between spatially separated regions indicate lower confidence in their relative positioning. Thus, while the local structures of the two PDZ domains are predicted with high confidence, the PAE analysis indicates greater uncertainty in the global interdomain arrangement. This distinction is consistent with the moderate global pTM score and supports a cautious interpretation of the predicted alternative topology as a possible structural model rather than a direct structural determination of the transient misfolded intermediate.

Table S1. Global confidence metrics for the five AlphaFold 3 predictions.

Model	pTM	Ranking score	Recycles
1	0.53	0.62	10
2	0.52	0.61	10
3	0.52	0.61	10
4	0.52	0.60	10
5	0.52	0.60	10

All five models reproduced the alternative topology described in the main text.
